# Editorial: Physical culture for mental health

**DOI:** 10.3389/fpsyg.2024.1537842

**Published:** 2025-01-06

**Authors:** Paweł Adam Piepiora, Zbigniew Norbert Piepiora, Daniela Stackeová, Justyna Bagińska, Bartłomiej Ga̧sienica-Walczak, Petra Čaplová

**Affiliations:** ^1^Faculty of Physical Education and Sports, Wroclaw University of Health and Sport Sciences, Wrocław, Poland; ^2^Faculty of Spatial Management and Landscape Architecture, Wroclaw University of Environmental and Life Sciences, Wrocław, Poland; ^3^Department of Pedagogy and Psychology, College of Physical Education and Sport Palestra, Prague, Czechia; ^4^Faculty of Humanities and Social Studies, Karkonosze University of Applied Sciences in Jelenia Gora, Jelenia Góra, Poland; ^5^Health Institute, University of Applied Sciences in Nowy Targ, Nowy Targ, Poland; ^6^Faculty of Sciences, Humanities and Education, Technical University of Liberec, Liberec, Czechia

**Keywords:** physical education, physiotherapy, recreation, sports, tourism

## Introduction

Physical culture encompasses all activities aimed at maintaining health through physical activity. This can be implemented in five distinct areas: physical education, physiotherapy, recreation, sports and tourism. Such physical activity is indicative of physical fitness, which is a contributing factor to overall health. Prior research in the field of physical culture has concentrated on the topic of bodily health. The positive bodily effects of physical culture, including the development of motor potential, the correction of postural defects, the maintenance of normal body weight, the control of neuromuscular tension, and active recreation, have been repeatedly emphasized in previous research. To a lesser extent, research in the physical culture sciences has addressed the significant psychosomatic effects of physical activity, such as stress relief, self-discipline, and redirecting negative habits toward competitive sport training, the positive impact of sport on personality formation, and more. However, there is a notable research gap regarding the role of physical activity in supporting mental health. This prompted the development of the Research Topic “*physical culture for mental health”*.

## The importance of physical culture for mental health

The acknowledgment of the role of psychiatric, psychological and psychotherapeutic interventions in the maintenance of mental health is a well-established concept in the field of psychiatry and psychology. Nevertheless, the importance of promoting, maintaining and restoring mental wellbeing through physical culture is often overlooked. The role of physical culture in supporting psychiatric, psychological and psychotherapeutic interactions is of significant importance. It serves to prevent and treat disorders while maintaining mental health. Furthermore, the field of physical culture sciences is situated within the domain of medical and health sciences.

The objective of this Research Topic is to present contemporary research exploring the impact of physical activity, encompassing physical education, physiotherapy, recreation, sports, and tourism, on mental health. The objective of this initiative is to disseminate research in the field of physical culture sciences, with a particular focus on mental health, which is of paramount importance in the context of the ongoing challenges that test our mental resilience. Regular physical activity is widely recognized as vital for maintaining mental health. This Research Topic aims to provide evidence to support this assumption.

An interdisciplinary approach has been taken in order to disseminate research findings on the complex relationship between physical culture and mental health. It is encouraging to note the international contributions of the authors, who will contribute to the holistic wellbeing of individuals in our society through the dissemination of their research achievements.

## Contents of the Research Topic

The authors who contributed to this Research Topic reached insightful conclusions, which contributed to the expansion of knowledge regarding the efficacy of physical culture for mental health.

In the initial article, Lin C. et al. conducted a comprehensive evaluation of the efficacy of chronic exercise as an intervention for executive function in children. The findings indicated that chronic exercise interventions consistently demonstrated a positive impact on executive functions, regardless of the duration of the intervention, the frequency of the sessions, or the length of each session.

Then, Huo et al. conducted a systematic review and synthesis of the literature on the relationship between sports participation and adolescent suicide-related outcomes. The findings indicated that increased participation in sports was associated with a reduction in suicidal ideation and suicide attempts among adolescents.

Subsequently, Liu R. et al. examined the impact of physical activity on health behaviors, mental health and psychological resilience, with a particular focus on the moderating role of quality of life. It has been observed that the quality of life exerts a moderating influence on the relationships between physical exercise and a number of factors, including psychological resilience, mental health and health-related behavior.

In a further work, Leung et al. conducted a pilot study with the objective of improving metabolic syndrome (MetS) screening, identification and intervention. This was achieved by offering streamlined lifestyle education, clinical reviews and discharge planning. The results demonstrated the urgent need for physical consumer-centered care and cultural change to promote collaboration within psychiatric services.

In a separate study, Lin L. et al. corroborated the correlations between compliance with 24-h exercise directives and mental wellbeing and quality of life among young adults, and delineated the mediating or moderating function of e-health utilization in these correlations. The findings indicated that adherence to a greater number of the guidelines was associated with enhanced mental health and quality of life among Chinese young adults. Conversely, meeting different combinations of the guidelines resulted in varying health outcomes.

In a subsequent study, Guo et al. verified the relationship between physical activity and subjective wellbeing among students and investigated the mediating role of social support and self-efficacy between physical activity and subjective wellbeing. It was determined that, in addition to encouraging physical activity, it is essential to prioritize the enhancement of social support and self-efficacy when promoting college students' subjective wellbeing.

In another study, Sheng et al. investigated the cross-sectional association between sports participation and resilience in children and adolescents as a means of enhancing mental health. The results of the cross-sectional study indicated a positive correlation between sports participation and resilience in children and adolescents, thereby highlighting the potential of encouraging sports participation as a strategy for promoting mental health resilience.

After that, Wrześniewski et al. sought to determine the optimal frequency, duration, and position of vibration treatments in relation to the reduction of subjectively perceived exertion, muscle pain, mental discomfort, emotional states, and the level of cognitive processes disturbed by intense physical activity. From a psychological perspective, the findings indicated that 10-min treatments at lower frequencies (in the range of 2–52 Hz) were more effective.

In the following article, Kruszewski et al. investigated the evolution of hand-to-hand exercises in Western culture, contrasting the traditional and modern forms. It has been demonstrated that only through the implementation of comprehensive education, coupled with a multifaceted programme of social role modeling and social facilitation, can a robust counterbalance to the growing prevalence of pathology in our lives be achieved. This, in turn, can lead to the promotion of positive social behaviors and the prevention of aggression and threats to physical and mental health.

Later, Hong et al. conducted a cross-sectional study to examine the relationship between physical fitness and mental health in a sample of Chinese college students. The evidence indicated a correlation between physical fitness and mental health and sleep quality among college students.

In a further contribution to the field, Fraile-Martinez et al. conducted a narrative review to investigate the underlying mechanisms linking resistance training to academic performance. In general, resistance training can have a beneficial impact on academic performance in children, adolescents and university students through various mechanisms. However, certain groups may derive greater benefits from its implementation, including individuals with inadequate training (particularly females) or those with psychosocial disorders, overweight or obesity.

Subsequently, Borysiuk et al. elucidated the structure of muscular activity and ground reaction forces during the preparatory period and the execution of a fencing lunge at the opponent's torso. The study demonstrated that the EMG signal was activated in advance of the generation of vertical force waveforms by a platform in response to ground forces within a 15–30 ms range. Furthermore, the importance of the preparation period for the effectiveness of the fencing stroke was emphasized based on the anticipatory phenomenon.

Furthermore, Zhang et al. investigated the relationship between various factors associated with sport anxiety (somatic anxiety, worry, and concentration disruption) and subjective happiness. Additionally, they examined the mediating role of need satisfaction in this relationship. The findings of this study not only provide further insight into the relationship between sport anxiety and subjective happiness but also offer new perspectives on mental health intervention programmes and strategies in college physical education.

Later, Deng and Wang present novel approaches to addressing social anxiety among college students by examining the potential mediating influence of social support and psychological capital in the relationship between physical activity and social anxiety. It was posited that physical activity can not only directly reduce social anxiety among college students, but also indirectly alleviate social anxiety among college students by increasing the level of social support and psychological capital.

A subsequent study by Piepiora and Gwardyński presents a novel argument in favor of the efficacy of karate as a tool for youth safety education. It has been demonstrated that karate is an efficacious method for educating young people in matters of safety. However, for this approach to be effective, it must be implemented in its entirety. The combat sport perspective engages young people in physical culture, the martial art perspective educates according to established norms and rules, and the self-defense system perspective teaches how to respond effectively in challenging situations.

Also, Liu C. et al. conducted a narrative review of the specific theoretical and practical experiences of parents in sport parenting. This was based on an analysis of the types and functions that constitute parental involvement in sport parenting, as well as an examination of the processes involved in their practice. It is assumed that parents, when participating in sports parenting, must regulate their own behavior in order to avoid placing psychological pressure on their children due to inappropriate behavior; assume different roles at different stages of their children's sports development; and avoid placing excessive pressure on their children's performance.

In the subsequent article, Piepiora, Bagińska et al. highlighted the declining interest in physical activity from the perspective of inadequate communication of physical culture to contemporary generations. The cohort designated as “Baby Boomers” (BB) is followed by the “Great Unknowns” (X), the “Millennials” (Y), and the “Snowflakes” (Z). The final group is the “Alpha” generation, which is defined by its digital orientation. It was concluded that the activation of these social groups should be conducted by addressing topics that are of importance to them.

Then, Kang and Gong investigated their mediating role in the relationship between maladaptive perfectionism and athlete burnout. The relationship between maladaptive perfectionism and athlete burnout can be partially explained by the mediating role of individuals' fear of failure and self-handicapping behaviors.

Moreover, Liu L. et al. examined the relationship between yoga exercise intervention and the comprehensive wellbeing of college students, as well as the underlying mechanisms through which yoga exercise intervention may enhance the comprehensive wellbeing of female college students. The findings indicated that yoga exercise intervention was associated with improved comprehensive wellbeing in female college students, suggesting that it may serve as an effective counseling method for promoting mental health in this population.

Subsequently, Piepiora, Caplová, Zimoń et al. investigated the correlation between personality traits and the competitive level of athletes. It was determined that the observed discrepancies between the examined athlete groups could be attributed to the distinctive characteristics of the respective sports. The elite cohort exhibited a reduced intensity of neuroticism in comparison to the competitive athletes.

In the next article, Sui et al. investigated the impact of physical exercise on the perception of social fairness among college students, with a particular emphasis on the potential mediating role of perceived social support and life satisfaction. The researchers concluded that physical exercise has a positive effect on both perceived social support and the sense of social fairness. Furthermore, the impact of physical exercise on the perception of social fairness is both direct and indirect.

After that, Piepiora, Caplová, and Cynarski proposed that the incorporation of karate into the domain of physical culture represents a contemporary and appealing physical activity. It was concluded that karate is present in all areas of physical culture, including as a combat sport, martial art, and self-defense system. However, practicing karate only as a martial art provides opportunities for the maintenance of health.

Other authors: Li et al. conducted a narrative review to investigate the potential of physical activity as an intervention for adolescent psychological stress management and mental health promotion. The review identified effective implementation strategies and multi-sector collaboration as crucial for optimizing the benefits of physical activity interventions. In a subsequent study, Jalowska et al. investigated the relationship between coping with stressful situations, the level of aerobic capacity, and post-workout restitution. Additionally, they examined the changes that occur between these variables throughout the training camp preceding international men's championship competitions in classical-style wrestling in the age category cadet. Significant correlations were identified between the style of coping with stress and scores in the performance test and the number of heart contractions in athletes preparing for the European Cadet Men's Wrestling Championships.

Next, Ambroży et al. sought to investigate how disparate demographic groups conceptualize success in martial arts. The study indicated that there was a weak correlation between the type of martial art practiced and the perception of success. A weak correlation was observed between nationality and the perception of success. A strong correlation was identified between age and perceived success, and a significant correlation was also observed between sex and perceived success.

In the subsequent article, Wang et al. sought to investigate the potential mediating role of negative physical sensation and experiential avoidance in the association between endurance exercise and exercise anxiety among university students. The findings indicated that endurance exercise affects exercise anxiety through two pathways: (1) endurance exercise → negative physical sensation → exercise anxiety; and (2) endurance exercise → negative physical sensation → experiential avoidance → exercise anxiety. Negative physical sensations and experiential avoidance serve as mediators and chain mediators between endurance exercise and exercise anxiety.

In a further study, Cao et al. employed a cross-sectional design to investigate a sample of Chinese undergraduate female college students using the Physical Activity Rating Scale, College Mental Health Scale, Brief Coping Styles Questionnaire, and Psychological Resilience Scale. The study demonstrated the underlying mechanisms through which physical exercise predicts mental health levels among female college students.

Then, Guo et al. examined the mediating effects of social interaction and emotional experience, offering insights for fostering greater participation in sports events and supporting the healthy development of the sports industry. It was concluded that viewing sporting events can act as a catalyst for enhancing wellbeing, primarily through fostering social connections and enriching emotional experiences.

In the penultimate article, Petre et al. conducted a systematic review to synthesize the evidence on the efficacy of internet- and mobile-based interventions for post-acute care in these conditions. Internet- and mobile-based interventions have demonstrated promise for aftercare and relapse prevention in depression, with limited evidence for anxiety disorders.

In the concluding article, Sarikabak et al. examine the relationship between conscientiousness and emotional intelligence in Paralympic athletes. It was proposed that the promotion of emotional intelligence in Paralympic athletes may serve to enhance their conscientiousness, thereby conferring benefits in terms of resilience and psychological stability.

